# Comparison of the long‐term outcomes of patients with hepatocellular carcinoma within the Milan criteria treated by ablation, resection, or transplantation

**DOI:** 10.1002/cam4.5063

**Published:** 2022-08-25

**Authors:** Ning‐Ning Zhang, Jian Zheng, Ying Wu, Jia‐Yu Lv, Shu‐Wen Zhang, Ya‐Min Zhang, Wen‐Tao Jiang, Tian‐Qiang Song, Victoria Kim, Samer Tohme, Tian Liu, Wei Zhang, Jie Gu, Ze‐Yu Wang, Yu‐Hong Suo, Shuai Wang, Wang Li, Li Zhang, Yan Xie, Yong‐He Zhou, Jian‐Yong Liu, Yi‐Bo Qiu, Zhong‐Yang Shen, Ji‐Hui Hao, David Geller, Wei Lu

**Affiliations:** ^1^ Department of Hepatobiliary Oncology, Liver Cancer Center, National Clinical Research Center for Cancer, Key Laboratory of Cancer Prevention and Therapy, Tianjin’s Clinical Research Center for Cancer Tianjin Medical University Cancer Institute and Hospital, Tianjin Medical University Tianjin China; ^2^ Post‐Doctoral Research Center Nankai University Tianjin China; ^3^ Department of Hepatobiliary Surgery, Tianjin First Central Hospital, Tianjin Key Laboratory for Organ Transplantation Tianjin China; ^4^ Department of Surgery University of Pittsburgh Medical Center Pittsburgh Pennsylvania USA; ^5^ School of Statistics and Data Science Nankai University, Key Laboratory for Medical Data Analysis and Statistical Research of Tianjin Tianjin China; ^6^ Department of Hepatology The Third Central Hospital of Tianjin Tianjin China; ^7^ Department of Liver Transplantation, Tianjin First Center Hospital, NHC Key Laboratory for Critical Care Medicine, Key Laboratory of Transplantation, Chinese Academy of Medical Sciences Tianjin China; ^8^ Department of Hepatobiliary Surgery, Liver Cancer Center Tianjin Medical University Cancer Institute and Hospital National Clinical Research Center for Cancer, Key Laboratory of Cancer Prevention and Therapy, Tianjin's Clinical Research Center for Cancer, National Human Genetic Resources Sharing Service Platform Tianjin Medical University Tianjin China; ^9^ Tianjin Second People’s Hospital, Tianjin Medical Research Institute of Liver Disease Tianjin China; ^10^ Department of Pancreatic Cancer Tianjin Medical University Cancer Institute and Hospital National Clinical Research Center for Cancer, Key Laboratory of Cancer Prevention and Therapy Tianjin China

**Keywords:** ablation, hepatocellular carcinoma, liver transplantation, Milan criteria, prognostic calculator, resection

## Abstract

**Background:**

Liver transplantation (LT), resection (LR), and ablation (LA) are three curative‐intent treatment options for patients with early hepatocellular carcinoma (HCC). We aimed to develop a prognostic calculator to compare the long‐term outcomes following each of these therapies.

**Methods:**

A total of 976 patients with HCC within the Milan criteria who underwent LT, LR, and LA between 2009 and 2019 from four institutions were evaluated. Multistate competing risks prediction models for recurrence‐free survival (RFS), recurrence within the Milan criteria (RWM), and HCC‐specific survival (HSS) were derived to develop a prognostic calculator.

**Results:**

During a median follow‐up of 51 months, 420 (43%) patients developed recurrence. In the multivariate analysis, larger tumor size, multinodularity, older age, male, higher alpha‐fetoprotein (AFP), higher albumin‐bilirubin (ALBI) grade, and the presence of portal hypertension were significantly associated with higher recurrence and decreased survival rates. The RFS and HSS were both significantly higher among patients treated by LT than by LR or LA and significantly higher between patients treated by LR than by LA (all *p* < 0.001). For multinodular HCC ≤3 cm, although LT had better RFS and HSS than LR or LA, LA was noninferior to LR. An online prognostic calculator was then developed based on the preoperative clinical factors that were independently associated with outcomes to evaluate RFS, RWM, and HSS at different time intervals for all three treatment options.

**Conclusions:**

Although LT resulted in the best recurrence and survival outcomes, LR and LA also offered durable long‐term alternatives. This prognostic calculator is a useful tool for clinicians to guide an informed and personalized discussion with patients based on their tumor biology and liver function.

## Graphical abstract

For patients with hepatocellular carcinoma (HCC) within the Milan Criteria (MC), three curative‐intent treatment options are liver transplantation (LT), resection (LR) and ablation (LA). This online prognostic calculator was developed based on the preoperative clinical factors that were independently associated with outcomes to evaluate and compare recurrence‐free survival (RFS), recurrence within the Milan criteria (RWM), and HCC‐specific survival (HSS) at different time intervals for all three treatment options. Patients who recurred within the MC are potentially eligible for salvage transplantation.

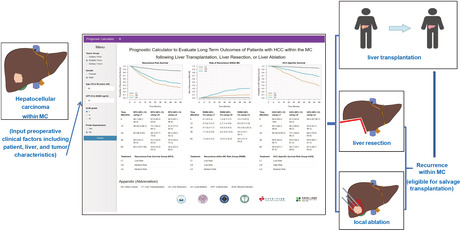

## 1 INTRODUCTION

Primary liver cancer is the fourth leading cause of cancer‐related death worldwide, and hepatocellular carcinoma (HCC) is the most common cause of primary liver cancer.^1^ Liver transplantation (LT), resection (LR), and ablation (LA) are the three potential first‐line curative options for patients with HCC within the Milan criteria (MC).^2^

Optimal treatment approach requires a comprehensive assessment of the patient's liver function and future liver remnant as well as patients' performance status and comorbidities.^2^ It is thus important that patients with HCC should be discussed in the multidisciplinary team (MDT) meetings in order to fully capture and create an individualized treatment plan according to clinical practice guidelines.^2^ However, the best treatment approach is often debated at the MDT meetings, as the optimal strategy should not only simply evaluate patients' tumor biology and liver function,^3^ but also consider local resources available for these three treatment modalities, as well as the feasibility of salvage LT in case of the recurrence.^4^ While many scoring systems have been developed to evaluate HCC prognosis, they focused on comparing one or two treatment modalities instead of all three curative options.^5^

The aim of this study was to compare the long‐term outcomes of LT, LR, and LA for patients with HCC within the MC and create an online prognostic calculator for their recurrence‐free survival (RFS), risk of recurrence within the MC (RWM), and HCC‐specific survival (HSS) using preoperative clinical data. This would help physicians guide patients in a personalized and informed discussion of their treatment options.

## 2 METHODS

### 2.1 Clinicopathologic characteristics

With the approval of the institutional review board at each center, we retrospectively identified 976 patients between 2009 and 2019 who had initial presentation of treatment‐naïve HCC within the MC at four medical centers. The Chinese cohort consisted of 716 patients who were then randomly assigned to the Chinese training cohort and the Chinese validation cohort in a 2:1 ratio to develop the three prognostic models for RFS, RWM, and HSS. These three models were then externally validated with the American cohort which consisted of 260 patients. For the Chinese cohort, we applied the simple random sampling method in the following steps. Firstly, we drew a random sample from the Chinese cohort to be in the training cohort by simple random sampling without replacement, for which each patient has equal probability of being selected. The sample size of this training cohort was approximately two‐third of the whole cohort. We used R function “*createDataPartition*” in the *caret* package to do this. Secondly, the remaining samples consisted of the validation cohort. Lastly, we further examined the balance between the training and validation cohorts; the details are shown in Table S1. Because of the property of simple random sampling technique, both the training and validation cohorts can provide unbiased representation of the whole cohort.

The study included adult patients who fulfilled all of the following inclusion criteria: (i) new diagnosis of HCC based on either imaging or histological evaluation according to the European Association for the Study of the Liver (EASL) guidelines^2^; (ii) Child–Pugh class A or B cirrhosis; and (iii) has HCC that is within the Milan criteria, defined as a solitary tumor ≤5 cm or 2–3 tumors, each ≤3 cm, without macrovascular invasion or extrahepatic metastasis.^6^

Patients were excluded if they had prior treatment for HCC, had Child–Pugh class C cirrhosis, or underwent LT, LR, or LA with non‐curative intent. Thus, patients who underwent LA while having additional untreated tumors as an attempt to bridge to LT were excluded. Patients who had concomitant LR, LA, and/or transcatheter arterial chemoembolization (TACE) were also excluded. All patients in this study underwent either LT, LR, or LA, which included either radiofrequency or microwave ablation.

In addition to comparing the long‐term outcomes of the three treatment groups (LT, LR, or LA) using RFS, RWM, and HSS, three additional subgroups under each treatment group were also further evaluated according to their tumor number and size: (i) solitary tumor ≤3 cm; (ii) multifocal tumors (2–3 tumors), each ≤3 cm; or (iii) a solitary tumor 3–5 cm.

The severity of liver disease was evaluated using the albumin‐bilirubin (ALBI) grade, Child–Pugh grade, aspartate aminotransferase‐to‐platelet ratio index (APRI) score, and Model for End‐Stage Liver Disease (MELD) score. Portal hypertension was diagnosed with indirect measures of ascites, and/or presence of gastroesophageal varices, and/or splenomegaly with a platelet count of less than 100 × 10^9^/L. Patients' actual treatments were decided generally based on the EASL guidelines^2^ after considering tumor size, number, location, liver function, future liver remnant, as well as patient comorbidities and functional status.

### 2.2 Surveillance and endpoints

Following LT, LR, or LA, patients were evaluated using α‐fetoprotein (AFP), liver function tests, and computed tomography during follow‐up every 3 months during the first 2 years and every 6 months thereafter. Recurrences were classified as within the MC or beyond the MC at the time of the first recurrence.

The primary endpoint of our study was to compare RWM, RFS, and HSS among patients who underwent LT, LR, or LA. RWM was the time from the date of treatment to the date of the first recurrence within the MC. Patients who recurred beyond the MC or were recurrence‐free at the date they were last known to be alive were censored at this time. RFS was defined as the time from treatment to recurrence or death, whichever was observed first. Patients were censored if they were alive without recurrence at the last visit. HSS was defined as the time of diagnosis to recurrence‐related death, and patients were censored if they were alive at the last visit or died from non‐HCC‐related causes.

### 2.3 Statistical analysis

Continuous variables were presented as medians and interquartile ranges, whereas categorical variables were presented as frequencies and proportions. Continuous variables in the training and validation cohorts were compared using Kruskal–Wallis tests. Categorical variables were compared using chi‐square tests or Fisher's exact tests, as appropriate. Nonparametric analyses of RFS and HSS for the three treatment groups were performed with Kaplan–Meier (K–M) estimators and compared with log‐rank tests. Competing risks analyses were conducted to estimate the cumulative incidence functions of the RWM and test equality across groups. In order to overcome the treatment selection bias in the Chinese training cohort, we performed the inverse probability of treatment weighting (IPTW) based on the method of the propensity score to evaluate the three options.

Univariate Cox PH models of RFS, RWM, and HSS were used to examine the association of the responses with each covariate separately. Since the three treatments were found to have different effects in the solitary and multiple HCC patients in our study, we developed multivariate Cox PH models including the interaction term between treatment allocation and tumor number. Stepwise regression was used to select the final model with the smallest Akaike information criterion (AIC). To model and predict the recurrence within the MC, we conducted a competing risks model and developed a multivariate cause‐specific Cox PH model based on stepwise selection, treating recurrence beyond the MC, and death for other than HCC as competing risks. To analyze the HSS, we performed another competing risk model for death due to HCC, treating non‐HCC‐related death as a competing risk.

To evaluate and validate model performance, discrimination abilities and calibration were evaluated for the Chinese training cohort, Chinese validation cohort, and American validation cohort. A receiver operating characteristic (ROC) curve and the concordance index were used to assess the discrimination, and a calibration plot comparing the predicted probability with the observed probability was used to assess the calibration ability. The risk scores were used to stratify patients into low‐, intermediate‐, and high‐risk groups. An online prognostic calculator to predict the RFS, the RWM, and the HSS was developed as an interactive web application using R Shiny App.^7^ The calculator allows users to easily adjust the values of significant preoperative clinical factors based on the developed models and view the predictive results in real time. Statistical analyses were performed using R version 3.5.3 (R Foundation for Statistical Computing, Vienna, Austria). *p* < 0.05 was used to indicate statistical significance and all statistical tests were two sided.

## 3 RESULTS

### 3.1 Clinicopathologic characteristics

In this study, 976 treatment naïve patients with early HCC within MC were included, of whom 716 were Chinese patients and 260 were American patients. They underwent LT (*n* = 311), LR (*n* = 312), or LA (*n* = 353) as their first‐line treatment (Table 1).

**TABLE 1:** Clinicopathologic characteristics of patients who underwent liver transplantation, resection, or ablation

Covariates	LT	LR	LA	*p* value
Patient number	311	312	353	
Gender (male)	270 (86.82%)	243 (77.88%)	261 (73.94%)	<0.001
Age (years)*	54.00 (46.50, 60.00)	57.50 (51.00, 66.00)	60.00 (54.00, 68.00)	<0.001
BMI (kg/m^2^)*	25.40 (23.16, 28.08)	24.88 (22.65, 27.21)	25.01 (22.86, 28.13)	0.321
Hypertension	81 (26.05%)	96 (30.77%)	143 (40.51%)	<0.001
Diabetes type 2	73 (23.47%)	66 (21.15%)	97 (27.48%)	0.154
ln(AFP) (ng/ml)*	2.43 (1.57, 4.14)	2.43 (1.46, 5.16)	2.22 (1.40, 3.71)	0.033
ln(PLT) (109/L)*	4.39 (3.92, 4.88)	5.06 (4.65, 5.30)	4.69 (4.29, 5.08)	<0.001
ln(ALB) (g/L)*	3.58 (3.47, 3.69)	3.74 (3.63, 3.82)	3.65 (3.50, 3.74)	<0.001
ln(AST) (U/L)*	3.75 (3.37, 4.15)	3.33 (3.09, 3.74)	3.64 (3.26, 4.06)	<0.001
ln(TBIL) (μmol/L)*	3.08 (2.79, 3.51)	2.84 (2.55, 3.00)	2.85 (2.80, 3.23)	<0.001
ln(Cr)(μmol/L)*	4.29 (4.13, 4.48)	4.34 (4.17, 4.48)	4.34 (4.13, 4.48)	0.598
ln(PT) (s)*	2.71 (2.59, 2.83)	2.45 (2.38, 2.57)	2.64 (2.56, 2.73)	<0.001
MELD score*	10.03 (7.76, 12.91)	7.47 (6.40, 8.48)	8.38 (7.05, 10.58)	<0.001
Child–Pugh grade				<0.001
A	183 (58.84%)	290 (92.95%)	274 (77.62%)	
B	128 (41.16%)	22 (7.05%)	79 (22.38%)	
ALBI score*	−2.14 (−2.57, −1.74)	−2.80 (−3.09, −2.42)	−2.42 (−2.76, −1.91)	<0.001
ALBI grade				<0.001
I	72 (23.15%)	203 (65.06%)	132 (37.39%)	
II	215 (69.13%)	103 (33.01%)	198 (56.09%)	
III	24 (7.72%)	6 (1.92%)	23 (6.52%)	
ln(APRI score)*	0.38 (−0.45, 1.06)	−0.66 (−1.21, −0.06)	−0.05 (−0.69, 0.64)	<0.001
Portal hypertension	243 (78.14%)	96 (30.77%)	275 (77.90%)	<0.001
Ascites	112 (36.01%)	22 (7.05%)	80 (22.66%)	<0.001
Cirrhosis	309 (99.36%)	229 (73.40%)	328 (92.92%)	<0.001
BCLC stage				0.259
0	72 (23.15%)	62 (19.87%)	89 (25.21%)	
A	239 (76.85%)	250 (80.13%)	264 (74.79%)	
Tumor subgroup				<0.001
Solitary ≤3 cm	155 (49.84%)	146 (46.79%)	243 (68.84%)	
Multiple ≤3 cm	88 (28.30%)	38 (12.18%)	40 (11.33%)	
Solitary 3–5 cm	68 (21.86%)	128 (41.03%)	70 (19.83%)	
Radiology HCC Number				<0.001
Solitary	222 (71.38%)	275 (88.14%)	313 (88.67%)	
Multiple	89 (28.62%)	37 (11.86%)	40 (11.33%)	
Radiology largest tumor diameter				<0.001
≤3 cm	244 (78.46%)	183 (58.65%)	283 (80.17%)	
3–5 cm	67 (21.54%)	129 (41.35%)	70 (19.83%)	

*Note*: Categorical variables were expressed in counts (proportions) and compared using Pearson's chi‐square or Fisher's exact test, as appropriate.

Abbreviations: AFP, α‐fetoprotein; ALBI, the albumin‐bilirubin; APRI, the aspartate aminotransferase‐to‐platelet ratio index; BCLC, Barcelona clinic liver cancer; BMI, body mass index; HCC, hepatocellular carcinoma; LA, liver ablation; LR, liver resection; LT, liver transplantation.

* Continuous variables were expressed in median values (interquartile range) and compared using Wilcoxon tests.

A total of 716 Chinese patients with HCC within the MC who underwent LT (*n* = 243), LR (*n* = 247), or LA (*n* = 226) as their first‐line treatments were randomly assigned to the Chinese training cohort (*n* = 482) and Chinese validation cohort (*n* = 234) in a 2:1 ratio. The long‐term outcomes of the training and validation cohorts were matched well in that the RFS, RWM, and HSS were not significantly different between them (*p* = 0.937, 0.786, and 0.977, respectively) (Figure S1). The American validation cohort was composed of additional 260 patients within the MC who also underwent LT (*n* = 68), LR (*n* = 65), or LA (*n* = 127). The clinicopathologic characteristics of the Chinese training cohort (*n* = 482), the Chinese validation cohort (*n* = 234), and the American validation cohort (*n* = 260) were summarized (Table S1).

### 3.2 Long‐term outcomes

During a median follow‐up of 50.8 months (IQR 36.3–77.5 months), 420 (43%) patients developed recurrence, of whom 245 (25.1%) experienced recurrence within MC, and 165 (16.9%) patients experienced recurrence beyond MC. Of 157 (44.5%) patients in the LA groups, only four patients underwent salvage transplantation. In contrast, only 10 (3.2%) patients in the LT group developed recurrence with the MC.

Among 243 transplanted patients in the Chinese training and validation cohorts, the median time spent on the waiting list in the LT group was 3.7 months, and 187 (77.0%) patients underwent bridging therapy prior to LT. This is in contrast to the American validation cohort, in which the median waitlist time was 8.4 months, and 67 (99%) underwent at least one bridging therapy prior to LT. This did not account for additional patients who had dropped out of the long waitlist for LT due to cancer progression or death.

In total, 309 (31.7%) patients died after a median follow‐up of 50.8 months, of which 244 (25%) patients' deaths were due to recurrence, and 65 (6.7%) patients' deaths were due to non‐HCC causes, which included graft malfunction, chronic rejection, acute liver failure, comorbidities, or other complications.

### 3.3 Development of the multistate competing risks prognostic models

Within the Chinese training cohort, clinicopathological variables among the LT, LR, and LA groups showed significant differences (Table S2). After the IPTW adjustment, the three treatment groups had similar baseline parameters including those deemed to potentially influence treatment selection, such as tumor size and number, AFP, and severity of underlying liver disease (Table S2). The RFS, RWM, and HSS among these three treatments in the Chinese training cohort before and after IPTW adjustment showed similar results (Figure S2).

In the Chinese training group, univariate analyses were performed to evaluate clinicopathological factors significantly associated with RFS, RWM, and HSS (Table S3). The multivariate analysis showed that older age, male gender, higher AFP, higher ALBI grade, larger tumor diameter (3–5 cm vs. ≤3 cm), multinodularity, and the presence of portal hypertension were prognostic for RFS, RWM, and HSS (Table 2).

**TABLE 2:** Multivariate analysis of the RFS, RWM, and HSS in the Chinese training cohort

Covariates	Recurrence‐free survival		Recurrence within Milan criteria		HCC‐specific survival	
	HR (95% CI)	*p* value	HR (95% CI)	*p* value	HR (95% CI)	*p* value
Age (years)	1.01 (1.00–1.03)	0.075				
Gender (male vs. female)	1.53 (1.05–2.23)	0.026			1.76 (1.03, 3.00)	0.039
In(AFP) (ng/ml)	1.08 (1.02–1.16)	0.014			1.10 (1.01, 1.20)	0.036
ALBI grade (II vs. I)	1.41 (1.04–1.91)	0.025	1.52 (1.03, 2.25)	0.034	1.81 (1.22, 2.69)	0.003
ALBI grade (III vs. I)	1.93 (1.04–3.59)	0.037	2.02 (0.89, 4.61)	0.093	3.42 (1.66, 7.04)	0.001
Portal hypertension	1.38 (0.96–1.99)	0.085	1.79 (1.09, 2.94)	0.022		
Operation						
LR vs. LT			12.75 (5.14, 31.59)	<0.001		
LA vs. LT			28.68 (12.32, 66.76)	<0.001		
LA vs. LR			2.25 (1.40–3.62)	<0.001		
Solitary tumor						
LR vs. LT	3.61 (2.05–6.35)	<0.001			7.49 (2.84–19.78)	<0.001
LA vs. LT	8.08 (4.80–13.61)	<0.001			20.42 (7.98–52.26)	<0.001
LA vs. LR	2.24 (1.50–3.35)	<0.001			2.73 (1.67–4.44)	<0.001
Multiple tumor						
LR vs. LT	6.87 (3.25–14.54)	<0.001			14.97 (5.78–38.80)	<0.001
LA vs. LT	4.92 (2.40–10.11)	<0.001			6.19 (2.31–16.63)	<0.001
LA vs. LR	0.72 (0.35–1.47)	0.362			0.41 (0.18–0.95)	0.038
Radiology HCC number (multiple vs. solitary)						
LT: Multiple vs. Solitary	1.73 (0.85–3.53)	0.131			3.81 (1.16–12.49)	0.027
LR: Multiple vs. Solitary	3.30 (1.80–6.05)	<0.001			7.62 (3.72–15.62)	<0.001
LA: Multiple vs. Solitary	1.05 (0.63–1.78)	0.841			1.16 (0.57–2.35)	0.686
Radiology largest tumor diameter (3–5 cm vs. ≤3 cm)	1.50 (1.09–2.07)	0.012	1.46 (0.98, 2.17)	0.064	2.16 (1.40, 3.34)	<0.001

*Note*: A multivariate cause‐specific Cox proportional hazards model was developed based on stepwise selection. A robust variance estimator was performed.

Abbreviations: AFP, α‐fetoprotein; ALBI, the albumin‐bilirubin; HCC, hepatocellular carcinoma; HSS, HCC‐specific survival; LA, liver ablation; LR, liver resection; LT, liver transplantation; RFS, recurrence‐free survival; RWM, recurrence within the Milan criteria.

**FIGURE 1:**
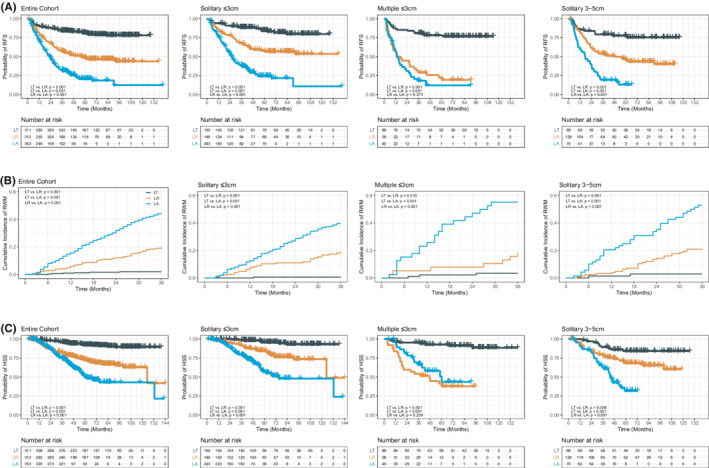
Comparison of RFS, RWM, and HSS among the three treatment groups. Comparison of RFS (A), RWM (B), and HSS (C) among the three treatment groups in the entire cohort and in different subgroups based on tumor number and size. HCC, hepatocellular carcinoma; HSS, HCC‐specific survival; LA, local ablation; LR, liver resection; LT, liver transplantation; RFS, recurrence‐free survival; RWM, recurrence within the Milan criteria.

In the subgroup analysis of tumors by size and multinodularity, for solitary tumors ≤3 cm or 3–5 cm, LT had significantly better RFS and HSS than LR or LA, and LR had significantly better RFS and HSS than LA (Table 2, Figure 1). However, for multinodular tumors (2–3 tumors, each ≤3 cm), although LT had significantly better RFS and HSS than LR or LA, the LA approach was noninferior to LR. (Table 2, Figure 1).

### 3.4 Internal and external validation of the RFS, RWM, and HSS models

**FIGURE 2:**
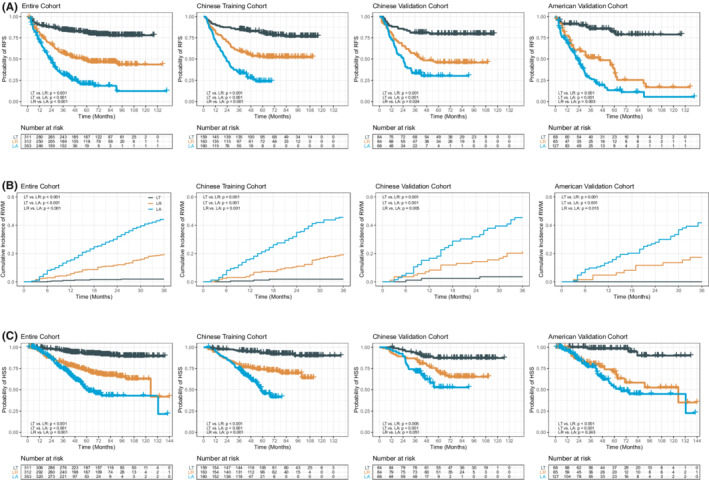
Comparison of RFS, RWM, and HSS among the three treatment groups in the training and validation cohorts. Comparison of RFS (A), RWM (B), and HSS (B) among the three treatment groups in the Chinese training cohort, Chinese validation cohort, and American validation cohort. HCC, hepatocellular carcinoma; HSS, HCC‐specific survival; LA, local ablation; LR, liver resection; LT, liver transplantation; RFS, recurrence‐free survival; RWM, recurrence within the Milan criteria.

The RFS, RWM, and HSS among LT, LR, and LA treatment groups showed similar results in the entire cohort as in the Chinese training cohort, the Chinese validation cohort, and the American validation cohort (Figure 2A–C).

In the entire cohort, the RFS and HSS were both significantly longer in patients treated by LT than LR or LA, and significantly longer in patients treated by LR than LA (all *p* < 0.001) (Figure 2A,C). This was consistent in the training and validation cohorts, with one exception is that the differences in HSS between LR and LA in the Chinese and American validation cohorts did not reach statistical difference (*p* = 0.051 and *p* = 0.243, respectively), which were likely due to smaller sample size.

For the cumulative incidence of RWM, since the recurrence rate following LT was extremely low, RWM was significantly higher in patients treated by LA or LR than LT, and significantly higher in patients treated by LA than LR (all *p* < 0.001) (Figure 2B). This finding applied to the entire cohort as well as the training and validation cohorts.

The risk scores based on the final multivariate models in the Chinese training cohort individually stratified the patients into three prognostic strata corresponding to low, intermediate, and high risks. Using −0.7 and 0.8, 0.2 and 1.1, −0.8 and 1 as the cutoff values for the RFS prediction model, the RWM prediction model, and the HSS prediction model, respectively. The low‐, intermediate‐, and high‐risk probabilities of RFS, RWM, and HSS based on the three models were well stratified among the entire cohort, Chinese training cohort, Chinese validation cohort, and American validation cohort (Figure S3).

### 3.5 Assessing model discrimination and calibration

The summary statistics c‐indexes of 2‐year RFS and RWM, 3‐year RFS, RWM, and HSS, and 5‐year RFS and HSS in the Chinese training cohort, Chinese validation cohort, and American validation cohort demonstrated the satisfactory predictive performance of these RFS, RWM, and HSS models (Table S4). The calibration plots of the model predicting 2‐year RFS and RWM (Figure S4A,B), 3‐year RWM (Figure S5C), 3‐year and 5‐year RFS (Figure S5A,B), and HSS (Figure S5D,E) in the Chinese training cohort, Chinese validation cohort, and American validation cohort further showed satisfactory agreement between the predicted and observed outcomes.

**FIGURE 3:**
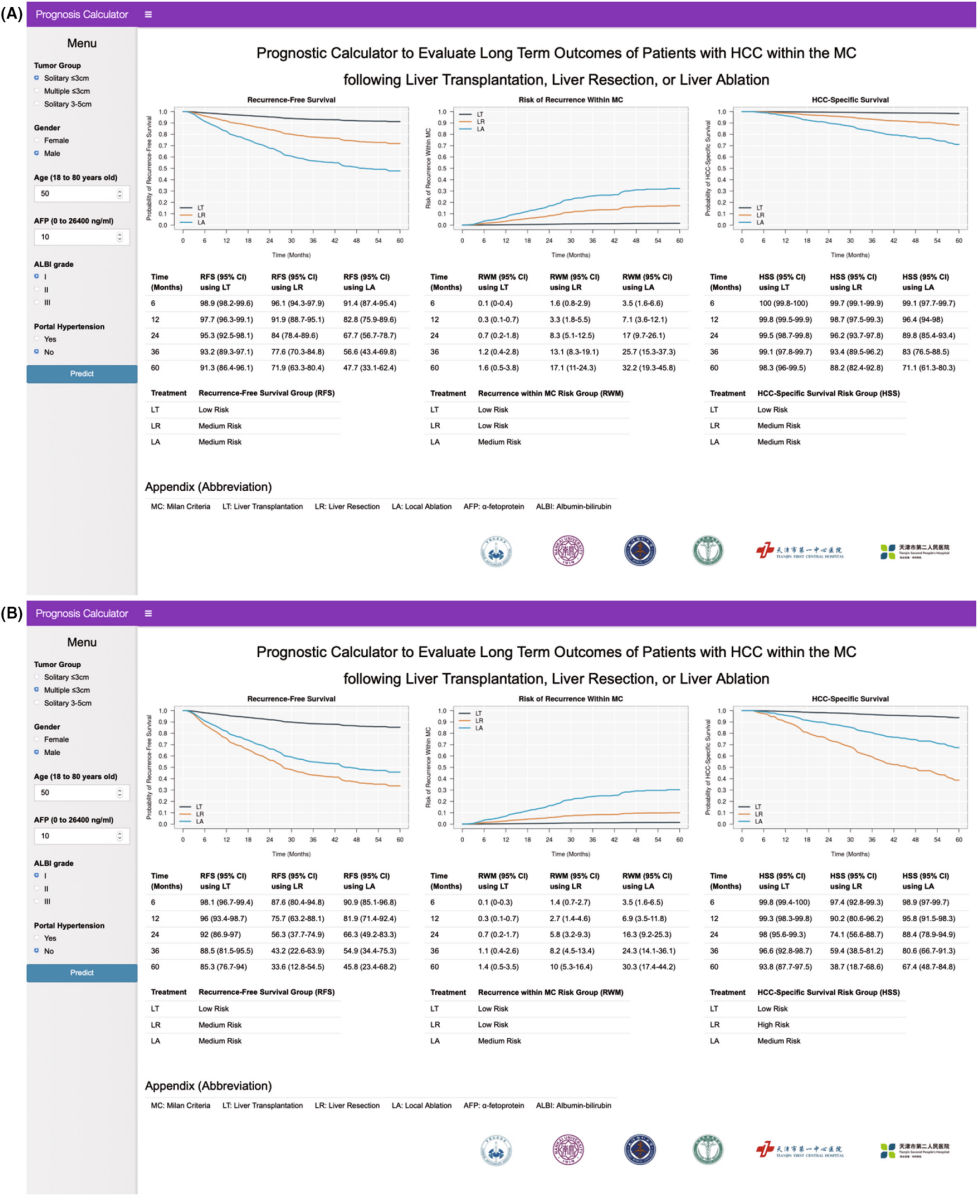
Prognostic online calculator to compare RFS, RWM, and HSS based on the treatment therapies for patients with HCC within the MC. The RFS, RWM, and HSS of a patient with solitary HCC ≤3 cm (A) versus multinodular HCC ≤3 cm (B). AFP, α‐fetoprotein; ALBI, albumin‐bilirubin; HCC, hepatocellular carcinoma; HSS, HCC‐specific survival; LA, local ablation; LR, liver resection; LT, liver transplantation; MC, Milan criteria; RFS, recurrence‐free survival; RWM, recurrence within the Milan criteria.

The prediction models of the RFS, the RWM, and the HSS were then used to create an online prognostic calculator: https://yingwu.shinyapps.io/Tianjin‐PC‐MDT/. By inputting preoperative clinical factors such as tumor size and number, gender, age, AFP level, ALBI grade, and portal hypertension status, the online calculator can reveal RFS, RWM, and HSS at different time intervals for all three treatment options. For example, in a 50‐year‐old male patient with a solitary HCC ≤3 cm, AFP 10 ng/ml, ALBI grade 1, and absence of portal hypertension, RFS, and HSS were higher when treated by LT than LR, and by LR than LA (Figure 3A) However, if the same patient had two or three multinodular HCC ≤3 cm instead of a single nodule, although LT was better than LR or LA, LA led to similar and better outcomes than LR in terms of RFS and HSS, respectively (Figure 3B).

## 4 DISCUSSION

In this study, 976 patients presented with treatment naïve early HCC within the Milan criteria underwent liver transplantation, resection, or ablation with curative intent and were followed to evaluate their recurrence‐free survival, recurrence within the Milan criteria, and HCC‐specific survival. Preoperative clinical factors, including tumor size and number, gender, age, AFP level, ALBI grade, and portal hypertension status, were independently associated with long‐term outcomes, consistent with prior studies.^8^, ^9^, ^10^, ^11^ Thus, these preoperative clinical factors were included to construct the online prognostic calculator, which would provide clinicians with a helpful tool to have an informed, individualized discussion with the patient by taking account of their tumor biology and liver function, as well as to portray their expected recurrence rate, recurrence pattern, and HCC‐specific survival over time.

After adjusting for clinically relevant preoperative factors, long‐term outcomes such as recurrence‐free survival and HCC‐specific survival were both significantly higher among patients treated by LT than by LR or LA and significantly higher in patients treated by LR than LA. LT may appear to be the most ideal treatment because of the improved long‐term outcomes from treating both the tumor and the underlying liver cirrhosis which is at risk of future recurrence or development of another de novo HCC. However, the use of LT has been limited by organ shortage, long transplant waitlist, dropout from progression or death, operative morbidities, as well as the need for lifelong immunosuppression.^12^, ^13^, ^14^ The median waiting times generally last over 12 months, with over one‐third of patients have dropped out from disease progression in a recent study.^15^ In addition, when intention to treat analysis was performed to include patients who had tumor progression or died while on the long waitlist, LT did not have survival benefit over LR.^16^, ^17^ Thus, the benefits and risks of LT, LR, or LA for individual patients based on their tumor biology, liver function, patient comorbidities, and functional are often debated in the multidisciplinary conferences.

Given the prognostic implications of these three treatment options as well as the limited resources available for LT, patients are often offered LR first if tumor is resectable with sufficient future liver remnant or otherwise offer LA if tumor is unresectable or patient cannot tolerate an operation.^18^, ^19^, ^20^ For LT, even if the liver organ is available for a patient with high MELD and relatively short waitlist as well as the ability to perform bridging therapies, patients to be eligible for LT still need to meet multiple stringent criteria, including age generally younger than 70, being physically fit, being abstinent of alcohol, and have a good support at home for a prolonged recovery and close follow‐up.^21^, ^22^ There are also differences to consider in these three treatment modalities in terms of associated surgical complications, length of stay, and cost, among other advantages and disadvantages of each treatment.^23^, ^24^

When offering these three first‐line treatment options, it is important to weigh in the morbidity of treatment, likelihood of recurrence, and patterns of recurrence within or beyond the MC, which will then dictate treatment options after recurrence. If patients underwent upfront liver resection or ablation but developed recurrence within the Milan criteria, they could theoretically be eligible for salvage transplantation, although the reality of its utilization was extremity low in our group, similar to prior studies because of the advanced age, acquired comorbidities, and scarcity of resources.^25^, ^26^ Cautions were also given because studies have also shown worse outcome following salvage transplantation than upfront transplantation given that recurrence following resection itself had already proven that HCC portended an aggressive tumor biology.^27^

In the subgroup analysis, while LR had significantly better RFS and HSS than LA for solitary tumors ≤3 cm or 3–5 cm, the LA approach was noninferior to LR for multinodular tumors (2–3 tumors, each ≤3 cm). One potential explanation is that multinodularity is a marker of an aggressive tumor biology, as it is associated with microvascular invasion, poor differentiation, and liver cirrhosis at risk for multiple de novo development of HCC or recurrence at the treated site. Because of this, the risk of recurrence would be high regardless of local LA or oncologic LR.^28^, ^29^ In this study, for multinodular HCC ≤3 cm, the median RFS following LR and LA were only 15.1 and 13.6 months, respectively (*p* = 0.373).

Microvascular invasion is a well‐known risk factor for recurrence following treatment of HCC.^28^, ^29^, ^30^ Microvascular invasion status was not built into our RFS, RWM, and HSS models because this online calculator was meant to include only preoperative clinical factors, whereas microvascular invasion status was obtained on pathological specimens. Although preoperative biopsy could obtain microvascular invasion status, it is rarely utilized given the risk of tumor seeding, poor concordance with final pathology, and because HCC is usually diagnosed by pathognomonic radiographic characteristics as described in the Liver Reporting & Data System (LI‐RADS).^31^ Most of the patients who underwent LA or LT with prior bridging therapy also lacked accurate MVI status.

Several prognostic systems have been developed to stratify patients with HCC, including the Barcelona Clinic Liver Cancer (BCLC) staging,^32^ Okuda staging,^33^ Cancer of the Liver Italian Program (CLIP) score,^34^ Japan Integrated Staging (JIS) Score,^35^ and the Chinese University Prognostic Index (CUPI).^36^ These prognostic systems are often used to guide management of all stages of HCC, but they do not provide detailed outcomes based on different treatment approaches for early HCC.^37^ Furthermore, an ideal prognostic model for risk stratification should not only be developed with appropriate methods but also be simple to use.^38^ Multiple prognostic nomograms have also been created in the past on individual treatment modality, but they are often difficult to use in everyday practice.^8^, ^39^ We constructed an easy‐to‐use online calculator by inputting preoperatively available clinical factors such as patient, tumor, and liver characteristics to predict different outcomes using each of the three potential treatment options for patients with early HCC. We provided RFS, RWM, and HSS with low‐, intermediate‐, and high‐risk stratifications. Based on reviewing this data, even if the long‐term outcomes for LTs were known to be better than LR and LA, physicians and patients may develop a shared decision to not enroll patient on the transplant waitlist because of other eligibility factors, estimated waiting time, availability of living donor program, personal preferences, and cultural beliefs, among other factors. Between LR and LA, LR may be favored over LA in the setting of solitary HCC given better outcomes with LR, but LA may be considered in the setting of multinodular HCC given their comparable long‐term results with LR. These shared decisions may also be affected by the availability of local resources and patient preference when taking account of these projected outcome differences.

While other research groups have evaluated the prognosis of patients with HCC, they typically focused on comparing one or two treatment modalities instead of evaluating all three curative options. To our knowledge, given scarce organ limitations of LT and ethical implications involved, there is no randomized controlled trial exists comparing all three modalities.^40^ We identified one prior retrospective study which was able to compare all three treatment modalities for solitary HCCs.^41^ They identified that among solitary HCC <3 cm, LA outcomes were similar to LR, and both were worse than LT. For solitary HCC 3 to 5 cm, LA outcomes were worse than LR, which were worse than LT. Thus, they concluded that RFA was not recommended for HCC larger than 3 cm, consistent with our and prior studies. We provided additional findings for multinodular HCC which were not examined in that study.

There are several limitations in this study. First, because of its retrospective and non‐randomized design, the baseline characteristics of the three treatment groups in the Chinese training cohort were different and thus may cause inherent biases when developing the prediction models. However, we did use IPTW adjustment to overcome these biases and got similar characteristics before and after the IPTW adjustments which improved the reliability of the prediction models. Second, although antiviral treatment was generally utilized for hepatitis B/C‐related HCC prior to these curative treatments, it was not included in our models because it was not documented in our databases. Third, because the majority of Chinese patients with HCC were related to hepatitis B cirrhosis (81.4% in the Chinese training and validation cohorts), they may present different tumor behaviors from the American patients with HCC as their cirrhosis was due to several other etiologies.^42^ The predominant causes of liver cirrhosis leading to HCC among the American validation cohort included hepatitis B (*N* = 8, 3.1%), hepatitis B + C (*N* = 4, 1.5%), hepatitis C (*N* = 128, 49.2%), alcohol use (*N* = 34, 13.1%), and non‐alcoholic steatohepatitis (*N* = 37, 14.2%). Thus, further studies especially multi‐institutional, prospective studies involving the global population would be required to further validate our model.

## 5 CONCLUSIONS

Preoperative clinical factors including tumor size and number, gender, age, AFP level, ALBI grade, and portal hypertension status were independently associated with recurrence‐free survival, recurrence within the Milan criteria, and HCC‐specific survival following liver transplantation, resection, or ablation. A prognostic online calculator was then created using these preoperative clinical factors to compare long‐term outcomes following these three first‐line treatment options. LT had better outcomes than LR and LA among all comers, but its utilization was limited by scarce organ availability. While the outcomes of LR were better than LA in patients with solitary HCC, their outcomes were comparable in patients with multinodular HCC. This online calculator is clinically useful, easy to use in everyday practice, and readily available online to guide clinicians in informed discussion of the optimal treatment strategies with patients and during multidisciplinary tumor board.

## AUTHORS' CONTRIBUTIONS

Concept and design: NNZ, JZ, DG, WL, JHH, YMZ, WTJ, TQS. Data collection: JZ, VK, JYL, JG, YHS, SWZ, TL, SW, WL, YBQ. Provision of study materials or patients: ST, ZYS, WZ, LZ, YX, YHZ, JYL. Statistical analysis: YW, NNZ, JZ, YMZ, WTJ, TQS, WL, JHH, DG. Writing of article: All authors.

## FUNDING INFORMATION

This work was supported by a grant from the Key Project of Science and Technology, Tianjin Science and Technology Committee (19YFZCSY00020).

## CONFLICT OF INTEREST

The authors report no conflicts of interest in this study.

## ETHICS APPROVAL

This study was conducted with the approval of the University of Pittsburgh (STUDY20090034), Tianjin Medical University Cancer Institute (bc2019082) and Hospital Medical Ethics Committee, Tianjin First Center Hospital Medical Ethics Committee (2016N070KY), and Tianjin Second People's Hospital Medical Ethics Committee, Tianjin, China (2016N043X).

## Supporting information


Appendix S1

## Data Availability

Data are available on request.
