# Supplementary material for: Comparison of the long‐term outcomes of patients with hepatocellular carcinoma within the Milan criteria treated by ablation, resection, or transplantation
Source: Cancer Med. 2022 Aug 25;12(3):2312–24. doi: 10.1002/cam4.5063 (PMC9939228; doi:10.1002/cam4.5063)
Supplement: Supplementary file 1 — Appendix S1 [file CAM4-12-2312-s001.doc]

**Supplementary material**

**Contents**

**1.Supplement tables**···········································································································P1-7

1.1. *Supplement Table 1*··································································································P1-2

1.2. *Supplement Table 2*··································································································P3-4

1.3. *Supplement Table 3*··································································································P5-6

1.4. *Supplement Table 4*······································································································P7

**2.Supplement figures**········································································································P8-12

2.1. *Supplement Fig.1*·······································································································P8

2.2. *Supplement Fig.2*·······································································································P9

2.3. *Supplement Fig.3*·····································································································P10

2.4. *Supplement Fig.4*·····································································································P11

2.5. *Supplement Fig.5*·····································································································P12

1. **Supplement tables：**

**Supplementary Table 1. Clinicopathologic Characteristics of the Chinese Training Cohort, Chinese Validation Cohort, and the American Validation Cohort**

| Covariates | Chinese Training | Chinese Validation | American Validation | *p* value |
| --- | --- | --- | --- | --- |
| Patient number | 482 | 234 | 260 |  |
| Operation |  |  |  | <0.0001 |
| LT | 159 (32.99%) | 84 (35.90%) | 68 (26.15%) |  |
| LR | 163 (33.82%) | 84 (35.90%) | 65 (25.00%) |  |
| LA | 160 (33.20%) | 66 (28.21%) | 127 (48.85%) |  |
| Gender (male) | 395 (81.95%) | 189 (80.77%) | 190 (73.08%) | 0.0142 |
| Age (years)^⁎^ | 55.50 (48.00, 62.00) | 55.00 (48.00, 60.00) | 65.00 (59.00, 74.00) | < 0.0001 |
| BMI (kg/m^2^)^⁎^ | 24.30 (22.15, 26.78) | 24.30 (22.28, 26.22) | 28.10 (25.30, 32.00) | < 0.0001 |
| Hypertension | 100 (20.75%) | 43 (18.38%) | 177 (68.08%) | <0.0001 |
| Diabetes type 2 | 85 (17.63%) | 36 (15.38%) | 115 (44.23%) | <0.0001 |
| ln(AFP) (ng/ml)^⁎^ | 2.57 (1.48, 4.81) | 2.55 (1.51, 5.22) | 2.02 (1.39, 3.25) | < 0.0001 |
| ln(PLT) (10^9^/L)^⁎^ | 4.73 (4.18, 5.15) | 4.70 (4.18, 5.15) | 4.73 (4.35, 5.17) | 0.3162 |
| ln(ALB) (g/L)^⁎^ | 3.69 (3.56, 3.78) | 3.67 (3.50, 3.78) | 3.61 (3.47, 3.71) | < 0.0001 |
| ln(AST) (U/L)^⁎^ | 3.55 (3.18, 3.95) | 3.50 (3.19, 3.97) | 3.74 (3.32, 4.16) | 0.0002 |
| ln(TBIL) (umol/L)^⁎^ | 2.88 (2.58, 3.26) | 2.92 (2.52, 3.36) | 2.84 (2.84, 3.12) | 0.0044 |
| ln(Cr)(umol/L)^⁎^ | 4.22 (4.09, 4.37) | 4.21 (4.09, 4.36) | 4.48 (4.48, 4.54) | < 0.0001 |
| ln(PT) (s)^⁎^ | 2.59 (2.43, 2.72) | 2.57 (2.43, 2.71) | 2.67 (2.59, 2.78) | < 0.0001 |
| MELD score^⁎^ | 7.92 (6.75, 10.38) | 8.17 (6.84, 10.61) | 8.80 (7.47, 11.66) | < 0.0001 |
| Child-Pugh grade |  |  |  | 0.3782 |
| A | 368 (76.35%) | 173 (73.93%) | 206 (79.23%) |  |
| B | 114 (23.65%) | 61 (26.07%) | 54 (20.77%) |  |
| ALBI score^⁎^ | -2.59 (-2.92, -2.09) | -2.50 (-2.96, -1.86) | -2.25 (-2.67, -1.83) | < 0.0001 |
| ALBI grade |  |  |  | <0.0001 |
| I | 239 (49.59%) | 99 (42.31%) | 69 (26.54%) |  |
| II | 219 (45.44%) | 118 (50.43%) | 179 (68.85%) |  |
| III | 24 (4.98%) | 17 (7.26%) | 12 (4.62%) |  |
| ln(APRI score)^⁎^ | -0.19 (-0.94, 0.63) | -0.19 (-0.90, 0.62) | -0.02 (-0.74, 0.74) | 0.4506 |
| Portal hypertension | 307 (63.69%) | 150 (64.10%) | 157 (60.38%) | 0.6127 |
| Ascites | 127 (26.35%) | 51 (21.79%) | 36 (13.85%) | 0.0004 |
| Cirrhosis | 422 (87.55%) | 207 (88.46%) | 237 (91.15%) | 0.3307 |
| BCLC stage |  |  |  | <0.0001 |
| 0 | 78 (16.18%) | 36 (15.38%) | 109 (41.92%) |  |
| A | 404 (83.82%) | 198 (84.62%) | 151 (58.08%) |  |
| Tumor Subgroup |  |  |  | <0.0001 |
| Solitary ≤3 cm | 241 (50.00%) | 110 (47.01%) | 193 (74.23%) |  |
| Multiple ≤3 cm | 97 (20.12%) | 53 (22.65%) | 16 (6.15%) |  |
| Solitary 3-5 cm | 144 (29.88%) | 71 (30.34%) | 51 (19.62%) |  |
| Radiology HCC Number |  |  |  | <0.0001 |
| Solitary | 385 (79.88%) | 181 (77.35%) | 244 (93.85%) |  |
| Multiple | 97 (20.12%) | 53 (22.65%) | 16 (6.15%) |  |
| Radiology Largest Tumor Diameter |  |  |  | 0.0054 |
| ≤3 cm | 338 (70.12%) | 163 (69.66%) | 209 (80.38%) |  |
| 3-5 cm | 144 (29.88%) | 71 (30.34%) | 51 (19.62%) |  |
| Differentiation |  |  |  | 0.0225 |
| Well | 129 (26.76%) | 61 (26.07%) | 72 (27.69%) |  |
| Moderate | 155 (32.16%) | 85 (36.32%) | 137 (52.69%) |  |
| Poor | 38 (7.88%) | 22 (9.40%) | 15 (5.77%) |  |
| Missing | 160 (33.20%) | 66 (28.21%) | 36 (13.85%) |  |
| Microvascular invasion |  |  |  |  |
| No | 251 (52.07%) | 130 (55.56%) | 53 (20.38%) | <0.0001 |
| Yes | 71 (14.73%) | 38 (16.24%) | 91 (35.00%) |  |
| Missing | 160 (33.20%) | 66 (28.21%) | 116 (44.62%) |  |

Categorical variables were expressed in counts (proportions) and compared using Pearson’s Chi-square or Fish’s exact test, as appropriate. ^⁎^Continuous variables were expressed in median values (interquartile range) and compared using Wilcoxon tests. LT, liver transplantation; LR, liver resection; LA, liver ablation; BMI, body mass index; AFP, α-fetoprotein; ALBI, the albumin-bilirubin; APRI, the aspartate aminotransferase-to-platelet ratio index; BCLC, Barcelona Clinic Liver Cancer; HCC, hepatocellular carcinoma.

**Supplementary Table 2. Baseline Characteristics of the Training Cohort Before and After IPTW.**

| Covariates | Before IPTW | | | | After IPTW | | | |
| --- | --- | --- | --- | --- | --- | --- | --- | --- |
|  | LT | LR | LA | *p*  value | LT | LR | LA | *p*  value |
| Gender (male) | 142 (89.31%) | 132 (80.98%) | 121 (75.62%) | 0.006 | 124 (82.67%) | 121 (73.33%) | 121 (82.88%) | 0.056 |
| Age (years) | 51.56 (8.10) | 55.46 (10.01) | 58.27 (8.97) | < 0.001 | 54.41 (7.70) | 56.12 (9.11) | 56.12 (9.11) | 0.220 |
| BMI (kg/m^2^) | 24.97 (3.72) | 24.58 (3.26) | 24.43 (3.64) | 0.465 | 24.79 (3.45) | 24.06 (3.24) | 24.06 (3.24) | 0.149 |
| Hypertension | 24 (15.09%) | 27 (16.56%) | 49 (30.63%) | 0.001 | 30 (20.00%) | 27 (16.36%) | 31 (21.23%) | 0.520 |
| Diabetes type 2 | 29 (18.24%) | 21 (12.88%) | 35 (21.88%) | 0.103 | 38 (25.50%) | 45 (27.27%) | 25 (17.24%) | 0.091 |
| ln(AFP) (ng/ml) | 3.17  (2.12) | 3.64 (2.37) | 2.76 (1.82) | 0.011 | 2.97 (2.22) | 3.32 (1.94) | 3.32 (1.94) | 0.255 |
| ln(TBIL) (umol/L) | 3.20  (0.71) | 2.69 (0.45) | 2.94 (0.49) | < 0.001 | 2.95 (0.63) | 2.86 (0.49) | 2.86 (0.49) | 0.189 |
| ln(ALB) (g/L) | 3.58  (0.16) | 3.74 (0.12) | 3.65 (0.16) | < 0.001 | 3.63 (0.15) | 3.66 (0.15) | 3.66 (0.15) | 0.246 |
| ln(Cr)(umol/L) | 4.25  (0.26) | 4.28 (0.23) | 4.18 (0.22) | < 0.001 | 4.23 (0.24) | 4.20 (0.35) | 4.20 (0.35) | 0.628 |
| ln(AST) (U/L) | 3.85  (0.77) | 3.46 (0.63) | 3.59 (0.54) | < 0.001 | 3.63 (0.65) | 3.65 (0.73) | 3.65 (0.73) | 0.722 |
| ln(PLT) (10^9^/L) | 4.32  (0.66) | 5.01 (0.48) | 4.61 (0.59) | < 0.001 | 4.54 (0.61) | 4.65 (0.58) | 4.65 (0.58) | 0.196 |
| MELD score | 11.12 (4.10) | 7.51 (1.93) | 8.59 (2.68) | < 0.001 | 9.03 (3.76) | 8.58 (2.51) | 8.58 (2.51) | 0.393 |
| Child-Pugh grade |  |  |  | 0.001 |  |  |  | 0.131 |
| A | 86 (54.09%) | 159 (97.55%) | 123 (76.88%) |  | 112 (74.67%) | 134 (81.21%) | 104 (71.72%) |  |
| B | 73 (45.91%) | 4 (2.45%) | 37 (23.12%) |  | 38 (25.33%) | 31 (18.79%) | 41 (28.28%) |  |
| ALBI score | -2.16 (0.57) | -2.85 (0.44) | -2.46 (0.56) | < 0.001 | -2.38 (0.54) | -2.51 (0.53) | -2.51 (0.53) | 0.117 |
| ln(APRI score) | 0.44  (1.05) | -0.63 (0.88) | -0.11 (0.91) | < 0.001 | 0.00 (0.96) | -0.08 (1.06) | -0.08 (1.06) | 0.742 |
| Ascites | 73 (45.91%) | 11 (6.75%) | 43 (26.88%) | < 0.001 | 41 (27.33%) | 45 (27.44%) | 39 (26.90%) | 0.994 |
| Portal hypertension | 128 (80.50%) | 46 (28.22%) | 133 (83.12%) | < 0.001 | 104 (69.33%) | 105 (63.64%) | 103 (70.55%) | 0.374 |
| Radiology HCC Number |  |  |  | < 0.001 |  |  |  | 0.280 |
| Solitary | 105 (66.04%) | 144 (88.34%) | 136 (85.00%) |  | 119 (79.33%) | 135 (81.82%) | 126 (86.30%) |  |
| Multiple | 54 (33.96%) | 19 (11.66%) | 24 (15.00%) |  | 31 (20.67%) | 30 (18.18%) | 20 (13.70%) |  |
| Radiology Largest Tumor Diameter |  |  |  | < 0.001 |  |  |  | 0.559 |
| ≤3 cm | 121 (76.10%) | 93 (57.06%) | 124 (77.50%) |  | 104 (69.33%) | 123 (74.55%) | 103 (70.55%) |  |
| 3-5 cm | 38 (23.90%) | 70 (42.94%) | 36 (22.50%) |  | 46 (30.67%) | 42 (25.45%) | 43 (29.45%) |  |
| Tumor Subgroup |  |  |  | < 0.001 |  |  |  | 0.402 |
| Solitary ≤3 cm | 67 (42.14%) | 74 (45.40%) | 100 (62.50%) |  | 73 (48.67%) | 92 (56.10%) | 83 (56.85%) |  |
| Multiple ≤3 cm | 54 (33.96%) | 19 (11.66%) | 24 (15.00%) |  | 31 (20.67%) | 30 (18.29%) | 20 (13.70%) |  |
| Solitary 3-5 cm | 38 (23.90%) | 70 (42.94%) | 36 (22.50%) |  | 46 (30.67%) | 42 (25.61%) | 43 (29.45%) |  |

Categorical variables were expressed in counts (proportions) and compared using Pearson’s Chi-square or Fish’s exact test, as appropriate. Continuous variables were expressed in median values (interquartile range) and continuous variables in the treatment groups were compared using Wilcoxon tests. IPTW, inverse probability of treatment weights; BMI, body mass index; AFP, α-fetoprotein; ALBI, the albumin-bilirubin; APRI, the aspartate aminotransferase-to-platelet ratio index; LT, liver transplantation; LR, liver resection; LA, liver ablation.

**Supplementary Table 3. Univariate Analyses of RFS, RWM and HSS in the Chinese Training Cohort**

| Covariates | Recurrence-free Survival | | Recurrence within Milan criteria | | HCC-Specific Survival | |
| --- | --- | --- | --- | --- | --- | --- |
|  | HR(95% CI) | *p* value | HR(95% CI) | *p* value | HR(95% CI) | *p* value |
| Operation |  | 0.145 |  | 0.637 |  | 0.002 |
| LR vs LT | 1.30 (0.92, 1.83) | 0.138 | 0.85 (0.51, 1.40) | 0.522 | 1.88 (1.18, 3.01) | 0.008 |
| LA vs LT | 1.31 (0.97, 1.77) | 0.081 | 1.10 (0.74, 1.64) | 0.632 | 2.00 (1.33, 3.01) | <0.001 |
| Gender (male vs female) | 1.18 (0.82, 1.69) | 0.380 | 0.92 (0.59, 1.44) | 0.712 | 1.45 (0.86, 2.46) | 0.164 |
| Age (years) | 1.03 (1.01, 1.04) | <0.001 | 1.03 (1.01, 1.05) | 0.002 | 1.03 (1.01, 1.05) | 0.004 |
| BMI (kg/m^2^) | 1.01 (0.97, 1.04) | 0.779 | 0.99 (0.94, 1.04) | 0.708 | 0.98 (0.93, 1.03) | 0.483 |
| Hypertension | 1.40 (1.03, 1.89) | 0.032 | 1.09 (0.70, 1.69) | 0.709 | 1.49 (0.99, 2.23) | 0.054 |
| Diabetes type 2 | 1.07 (0.76, 1.51) | 0.705 | 0.86 (0.52, 1.41) | 0.540 | 1.05 (0.65, 1.67) | 0.850 |
| ln(AFP) (ng/ml) | 1.05 (0.99, 1.11) | 0.129 | 1.02 (0.94, 1.11) | 0.614 | 1.06 (0.98, 1.15) | 0.122 |
| ln(ALB) (g/L) | 0.65 (0.30, 1.43) | 0.287 | 0.83 (0.28, 2.41) | 0.726 | 0.29 (0.11, 0.82) | 0.019 |
| ln(Cr) (umol/L) | 0.86 (0.50, 1.49) | 0.591 | 0.78 (0.37, 1.62) | 0.504 | 0.83 (0.40, 1.76) | 0.631 |
| ln(PT) (s) | 0.64 (0.35, 1.17) | 0.150 | 0.77 (0.34, 1.75) | 0.530 | 1.11 (0.52, 2.37) | 0.792 |
| ln(PLT) (10^9^/L) | 1.02 (0.84, 1.25) | 0.824 | 0.98 (0.75, 1.29) | 0.892 | 1.17 (0.88, 1.55) | 0.287 |
| ln(AST) (U/L) | 0.98 (0.81, 1.18) | 0.815 | 0.93 (0.72, 1.20) | 0.571 | 1.17 (0.93, 1.47) | 0.169 |
| ln(TBIL) (umol/L) | 0.94 (0.75, 1.18) | 0.571 | 0.93 (0.69, 1.26) | 0.645 | 1.07 (0.80, 1.44) | 0.646 |
| MELD score | 0.96 (0.92, 1.00) | 0.044 | 0.93 (0.88, 0.99) | 0.017 | 0.96 (0.91, 1.02) | 0.157 |
| Child-Pugh grade (B vs A) | 0.94 (0.68, 1.29) | 0.696 | 0.72 (0.45, 1.14) | 0.156 | 1.10 (0.73, 1.66) | 0.658 |
| ALBI score | 1.11 (0.89, 1.38) | 0.369 | 1.02 (0.76, 1.37) | 0.905 | 1.37 (1.03, 1.83) | 0.031 |
| ALBI grade |  | 0.351 |  | 0.623 |  | 0.026 |
| II vs I | 1.14 (0.87, 1.50) | 0.330 | 1.12 (0.78, 1.61) | 0.530 | 1.27 (0.87, 1.84) | 0.210 |
| III vs I | 1.45 (0.82, 2.59) | 0.204 | 1.41 (0.65, 3.10) | 0.386 | 2.42 (1.26, 4.64) | 0.008 |
| ln(APRI score) | 0.98 (0.87, 1.11) | 0.768 | 0.98 (0.83, 1.15) | 0.770 | 1.02 (0.87, 1.21) | 0.794 |
| Portal hypertension | 1.47 (1.10, 1.95) | 0.009 | 1.77 (1.19, 2.64) | 0.005 | 1.21 (0.83, 1.77) | 0.312 |
| Ascites | 0.86 (0.63, 1.18) | 0.350 | 0.84 (0.55, 1.27) | 0.408 | 0.81 (0.53, 1.24) | 0.325 |
| Cirrhosis | 1.03 (0.69, 1.55) | 0.874 | 1.13 (0.65, 1.97) | 0.669 | 1.24 (0.70, 2.20) | 0.471 |
| BCLC stage (A vs 0) | 2.00 (1.30, 3.08) | 0.002 | 1.59 (0.94, 2.69) | 0.082 | 3.11 (1.52, 6.37) | 0.002 |
| Radiology HCC Number  (multiple vs solitary) | 1.17 (0.85, 1.61) | 0.338 | 0.82 (0.51, 1.32) | 0.415 | 1.38 (0.92, 2.09) | 0.124 |
| Radiology Largest Tumor Diameter  (3-5 cm vs ≤3 cm) | 1.21 (0.92, 1.60) | 0.181 | 1.15 (0.79, 1.68) | 0.475 | 1.61 (1.12, 2.31) | 0.010 |
| Differentiation |  | 0.002 |  | 0.027 |  | 0.002 |
| Moderate vs well | 1.62 (1.05, 2.50) | 0.028 | 1.62 (0.82, 3.20) | 0.163 | 2.63 (1.36, 5.07) | 0.004 |
| Poor vs well | 2.74 (1.56, 4.79) | <0.001 | 3.22 (1.37, 7.53) | 0.007 | 4.06 (1.82, 9.04) | <0.001 |
| Microvascular invasion | 1.56 (1.03, 2.35) | 0.035 | 0.71 (0.32, 1.59) | 0.409 | 1.40 (0.79, 2.49) | 0.246 |

Cox proportional hazards model was developed based on stepwise selection. LT, liver transplantation; LR, liver resection; LA, liver ablation; BMI, body mass index; AFP, α-fetoprotein; MELD, Model for End-Stage Liver Disease; ALBI, the albumin-bilirubin; APRI, the aspartate aminotransferase-to-platelet ratio index; BCLC, Barcelona Clinic Liver Cancer. RFS, recurrence-free survival; RWM, recurrence within Milan criteria; HSS, HCC-specific survival; HCC, hepatocellular carcinoma.

**Supplementary Table 4. Prognostic performance of the RFS, RWM and HSS models**

|  | | Chinese Training | | Chinese Validation | | American Validation | |
| --- | --- | --- | --- | --- | --- | --- | --- |
|  |  | AUC | 95%CI | AUC | 95%CI | AUC | 95%CI |
| 2-year | RFS | 0.777 | 0.734-0.821 | 0.676 | 0.601-0.751 | 0.741 | 0.676-0.805 |
|  | RWM | 0.807 | 0.759-0.855 | 0.766 | 0.691-0.842 | 0.759 | 0.689-0.828 |
| 3-year | RFS | 0.798 | 0.759-0.838 | 0.747 | 0.681-0.812 | 0.803 | 0.743-0.863 |
|  | RWM | 0.817 | 0.776-0.858 | 0.787 | 0.719-0.855 | 0.769 | 0.704-0.833 |
|  | HSS | 0.785 | 0.733-0.837 | 0.703 | 0.610-0.797 | 0.680 | 0.600-0.761 |
| 5-year | RFS | 0.852 | 0.814-0.890 | 0.793 | 0.730-0.856 | 0.841 | 0.775-0.906 |
|  | HSS | 0.857 | 0.815-0.898 | 0.730 | 0.647-0.814 | 0.716 | 0.640-0.792 |

The computation of AUC and its 95% confidence interval were conducted using the pROC package of R. AUC, the area under the ROC curve; CI, confidence interval; RFS, recurrence-free survival; RWM, recurrence within the Milan criteria; HSS, HCC-specific survival; HCC, hepatocellular carcinoma.

1. **Supplementary figures**

**
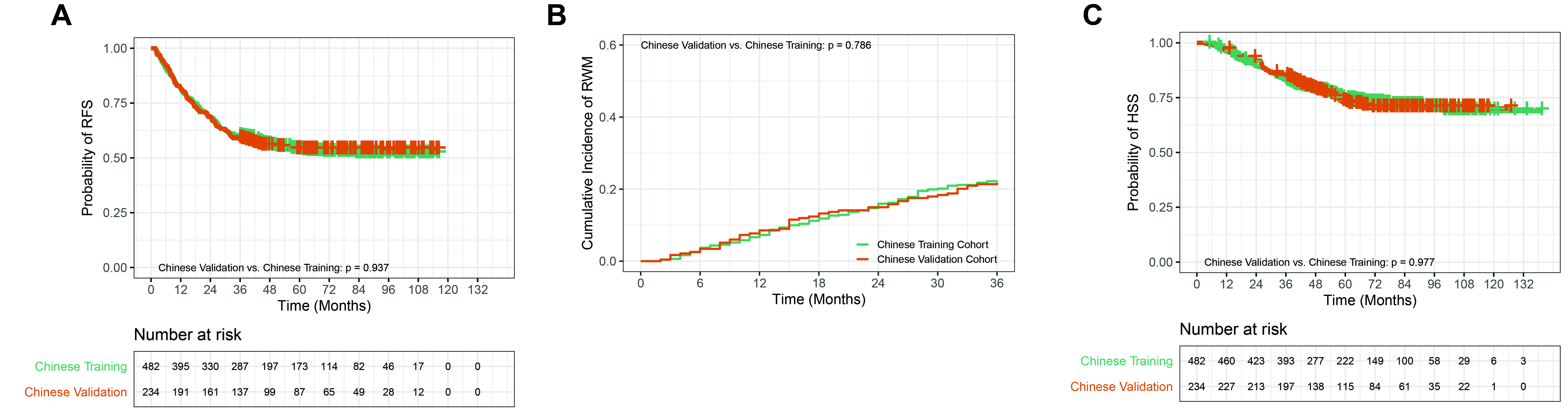
**

**Supplementary Fig. 1. Kaplan-Meier curves of** **RFS, RWM and HSS in the Chinese training cohort and Chinese validation cohort.** Kaplan-Meier curves of RFS (A), RWM (B) and HSS (C) in Chinese training cohort and Chinese validation cohort. RFS, recurrence-free survival; RWM, recurrence within the Milan criteria; HSS, HCC-specific survival; HCC, hepatocellular carcinoma.


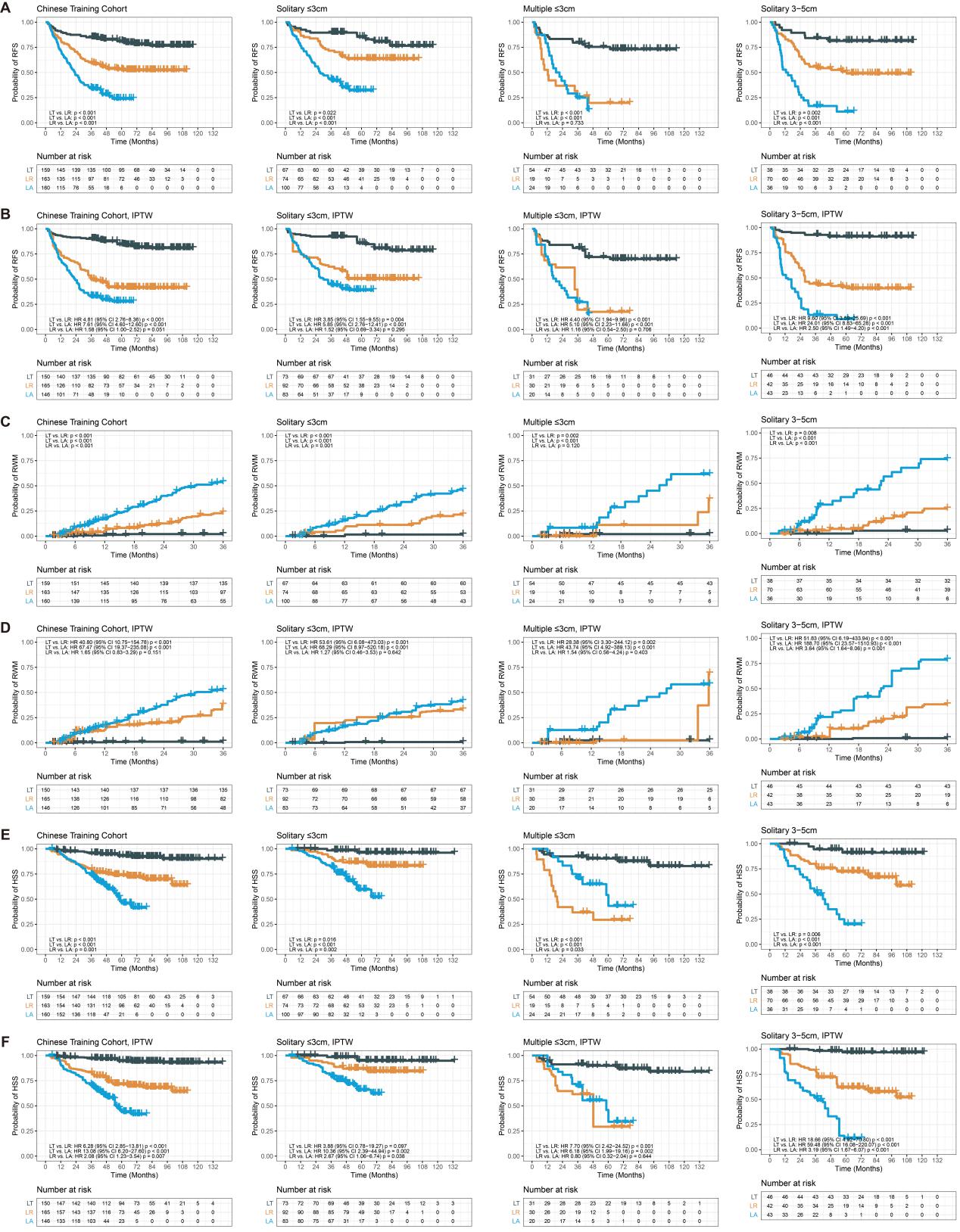
**Supplementary Fig. 2. Comparison of RFS, RWM and HSS among the three treatment groups and subgroups before and after IPTW in Chinese training cohort.** Comparison of RFS among the three treatment groups and subgroups before (A) and after (B) IPTW. Comparison of RWM among the three treatment groups and subgroups before (C) and after (D) IPTW. Comparison of HSS among the three treatment groups and subgroups before (E) and after (F) IPTW. RFS, recurrence-free survival; RWM, recurrence within the Milan criteria; HSS, HCC-specific survival; HCC, hepatocellular carcinoma; LT, liver transplantation; LR, liver resection; LA, local ablation; IPTW, inverse probability of treatment weighting.


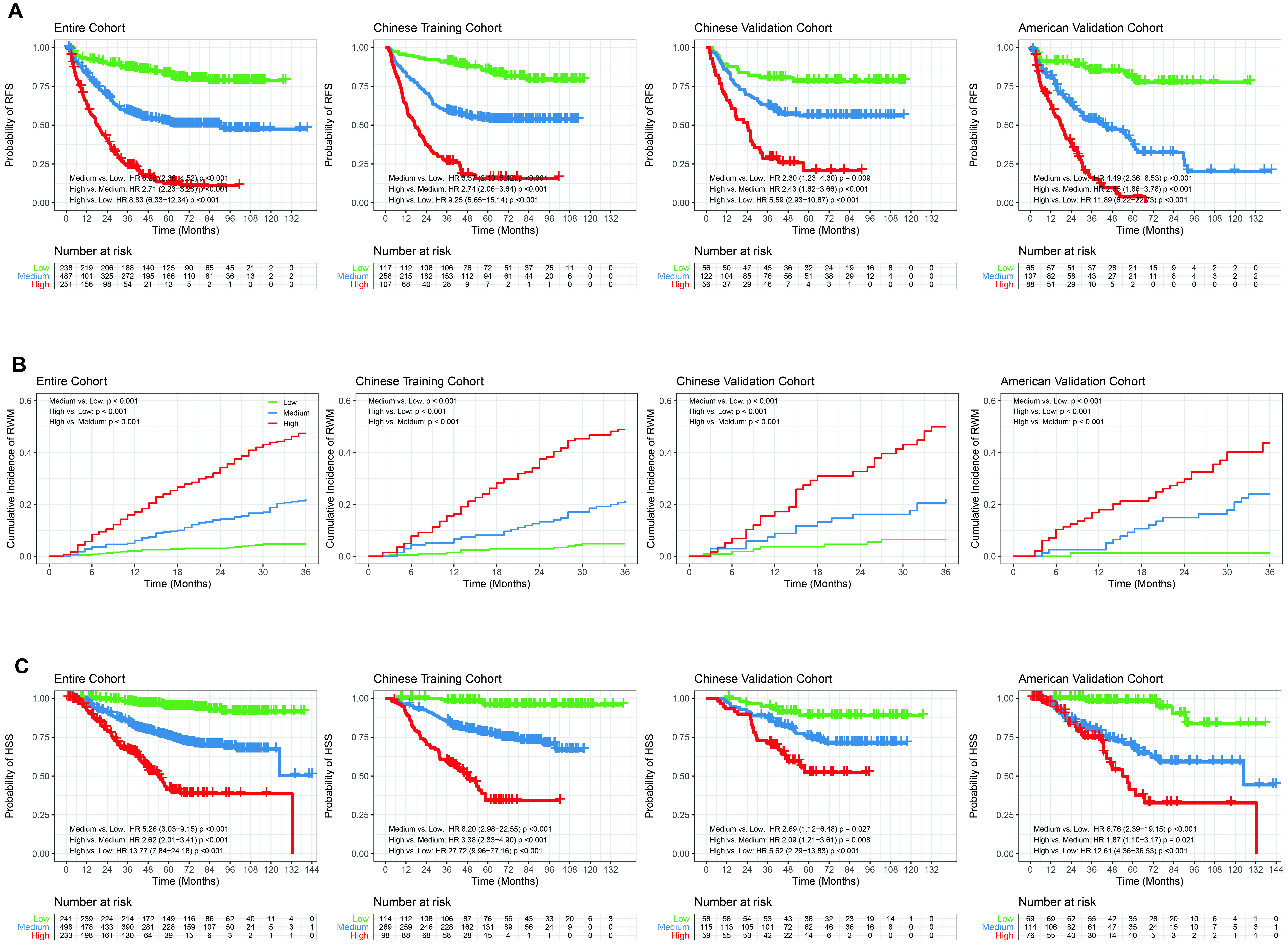
**Supplemental Fig. 3. Kaplan-Meier curves of RFS, RWM and HSS risk stratification based on three models.** Kaplan-Meier curves of low, intermediate, and high risks of RFS (A), RWM (B) and HSS (C). RFS, recurrence-free survival; RWM, recurrence within the Milan criteria; HSS, HCC-specific survival; HCC, hepatocellular carcinoma; HR, hazard ratio.


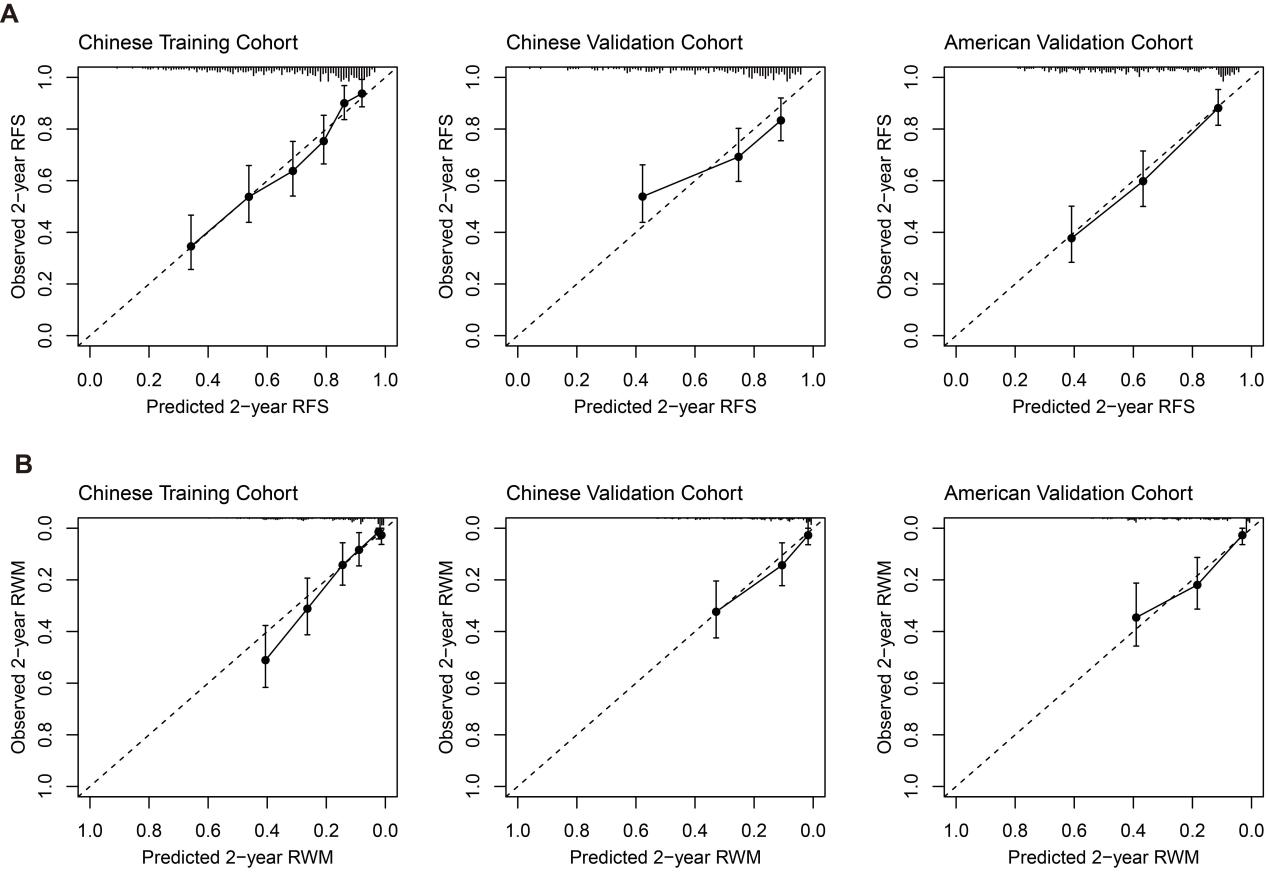
**Supplementary Fig 4. Calibration curves for the models in predicting 2-year RFS and 2-year RWM in the training cohort and validation cohort.** Calibration curves for the models in predicting 2-year RFS (A) and 2-year RWM (B) in training cohort and validation cohort. RFS, recurrence-free survival; RWM, recurrence within the Milan criteria.


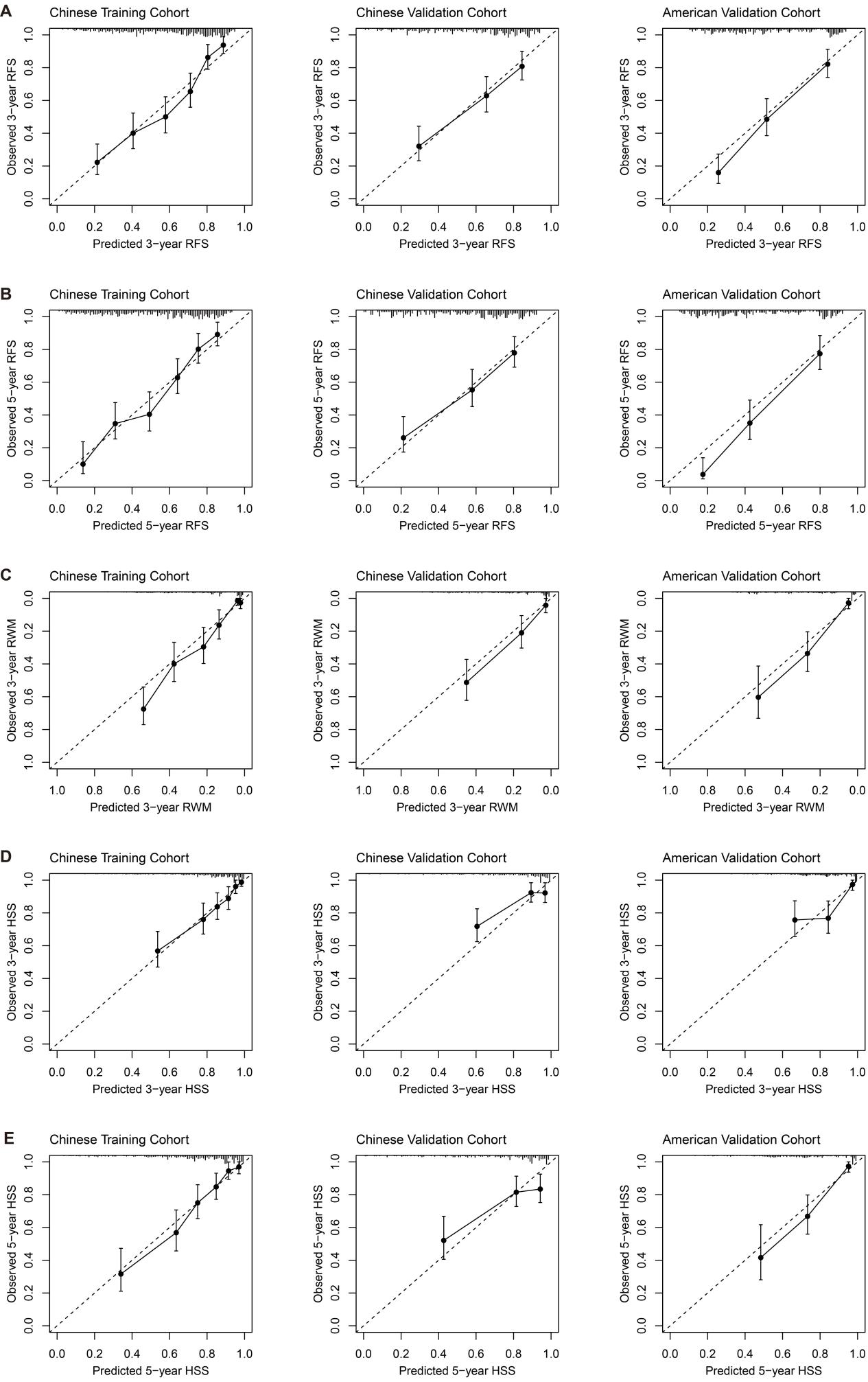
**Supplementary Fig. 5. Calibration curves for the models in predicting 3-year and 5-year RFS, 3-year RWM, and 3-year and 5-year HSS in the training cohort and validation cohort.** Calibration curves for the models in predicting 3-year (A) and 5-year (B) RFS in the training cohort and validation cohort. Calibration curves for the models in predicting 3-year RWM (C) in the training cohort and validation cohort. Calibration curves for the models in predicting 3-year (D) and 5-year (E) HSS in the training cohort and validation cohort. RFS, recurrence-free survival; RWM, recurrence within the Milan criteria; HSS, HCC-specific survival; HCC, hepatocellular carcinoma.
